# Upregulation of Immunoproteasome Subunits in Myositis Indicates Active Inflammation with Involvement of Antigen Presenting Cells, CD8 T-Cells and IFNγ

**DOI:** 10.1371/journal.pone.0104048

**Published:** 2014-08-06

**Authors:** Khetam Ghannam, Lorena Martinez-Gamboa, Lydia Spengler, Sabine Krause, Biljana Smiljanovic, Marc Bonin, Salyan Bhattarai, Andreas Grützkau, Gerd-R. Burmester, Thomas Häupl, Eugen Feist

**Affiliations:** 1 Department of Rheumatology and Clinical Immunology, Charité University Hospital, Berlin, Germany; 2 Friedrich Baur Institute, Ludwig Maximilians University, Munich, Germany; 3 German Arthritis Research Center, Berlin, Germany; National Institutes of Health, United States of America

## Abstract

**Objective:**

In idiopathic inflammatory myopathies (IIM) infiltration of immune cells into muscle and upregulation of MHC-I expression implies increased antigen presentation and involvement of the proteasome system. To decipher the role of immunoproteasomes in myositis, we investigated individual cell types and muscle tissues and focused on possible immune triggers.

**Methods:**

Expression of constitutive (PSMB5, -6, -7) and corresponding immunoproteasomal subunits (PSMB8, -9, -10) was analyzed by real-time RT-PCR in muscle biopsies and sorted peripheral blood cells of patients with IIM, non-inflammatory myopathies (NIM) and healthy donors (HD). Protein analysis in muscle biopsies was performed by western blot. Affymetrix HG-U133 platform derived transcriptome data from biopsies of different muscle diseases and from immune cell types as well as monocyte stimulation experiments were used for validation, coregulation and coexpression analyses.

**Results:**

Real-time RT-PCR revealed significantly increased expression of immunoproteasomal subunits (PSMB8/-9/-10) in DC, monocytes and CD8+ T-cells in IIM. In muscle biopsies, the immunosubunits were elevated in IIM compared to NIM and exceeded levels of matched blood samples. Proteins of PSMB8 and -9 were found only in IIM but not NIM muscle biopsies. Reanalysis of 78 myositis and 20 healthy muscle transcriptomes confirmed these results and revealed involvement of the antigen processing and presentation pathway. Comparison with reference profiles of sorted immune cells and healthy muscle confirmed upregulation of PSMB8 and -9 in myositis biopsies beyond infiltration related changes. This upregulation correlated highest with STAT1, IRF1 and IFNγ expression. Elevation of T-cell specific transcripts in active IIM muscles was accompanied by increased expression of DC and monocyte marker genes and thus reflects the cell type specific involvement observed in peripheral blood.

**Conclusions:**

Immunoproteasomes seem to indicate IIM activity and suggest that dominant involvement of antigen processing and presentation may qualify these diseases exemplarily for the evolving therapeutic concepts of immunoproteasome specific inhibition.

## Introduction

Idiopathic inflammatory myopathies (IIM) are a heterogenous group of muscle diseases characterized by inflammatory infiltrates in the skeletal muscle. Three major diseases are defined: dermatomyositis (DM), polymyositis (PM), and inclusion body myositis (IBM). The main autoimmune response in DM resembles a microangiopathy affecting skin and muscle tissue. In polymyositis and inclusion body myositis, cytotoxic CD8+ T cells invade muscle fibers. All of the invaded fibers and numerous non-invaded fibers express increased amounts of major histocompatibility complex class I (MHC-I) molecules [1]. In fact, the presence of MHC-I/CD8+ complex is considered as a characteristic immunopathological marker of PM and IBM [2]. Continuous upregulation of expression of MHC class I molecules in muscle fibers is thought to induce an endoplasmic reticulum stress response with accumulation of misfolded glycoproteins and activation of nuclear factor kappa B (NF-κB). As a consequence, MHC-I/CD8+ complexes may form and perpetuate an autoinflammatory response [3].

The ubiquitin-proteasome system (UPS) is a 26S, non-lysosomal, multicatalytic, and multisubunit complex involved in the ubiquitin-dependent, selective intracellular degradation of proteins [4]. In this way, the proteasome plays a central role in the activation of transcription factors such as NF-κB [5]. Furthermore, it is involved in the generation of peptides presented by the MHC-I to the antigen receptors of cytotoxic T cells [6], [7], and thus, is involved in the regulation of the inflammatory response. Many studies suggest that the proteasome participates in muscle fiber degradation in various physiological and pathological conditions and may therefore also play an important role in myositis [8], [9].

The 26S proteasome is composed of a proteolytically active core, namely the 20S proteasome, and one or two 19S regulator complexes. The 20S or constitutive proteasome is a cylindrical particle that consists of four rings, each composed of seven different subunits. The outer two rings are formed by seven alpha-type subunits (PSMA1–PSMA7), while the inner two rings contain seven beta-type subunits (PSMB1–PSMB7) [6]. The proteolytically active sites are limited to three constitutive beta subunits, proteasome subunit beta type 5-PSMB5, proteasome subunit beta type 6-PSMB6 and proteasome subunit beta type 7-PSMB7.[10]. Importantly, under the influence of the pro-inflammatory cytokine IFNγ, the structure and the catalytic properties of the constitutive proteasome are modified by substitution of the catalytic subunits PSMB5, PSMB6 and PSMB7 with three catalytic immunosubunits proteasome subunit beta type 8-PSMB8, proteasome subunit beta type 9-PSMB9 and proteasome subunit beta type 10-PSMB10 respectively, leading to the formation of the so-called immunoproteasome. This process is considered to strongly influence the production of peptides for antigen presentation by MHC class I as well as the immune response [11]–[13]. As IFNγ is also secreted in IIM [14], the immunoproteasomal system may also contribute to pathomechanisms in myositis. Recently, mutations in human were detected in proteasome subunit PSMB8, which cause joint contractures, muscle atrophy, microcytic anemia, and panniculitis-induced lipodystrophy syndrome in addition to other autoinflammatory syndromes [15]–[18]. Impaired immunoproteasome assembly and decreased proteolytic activity have been confirmed in some of these diseases. On the other hand, elevated levels of circulating proteasomes as well as autoantibodies against several proteasomal subunits have been detected in patients with autoimmune myositis and other autoimmune disorders [19], [20]. Moreover, it has also been shown that anti-proteasome autoantibodies derived from patients with connective tissue diseases, including a patient with polymyositis, were capable of blocking the stimulation of the catalytic proteasome core complex by the proteasome activator PA28, consisting of the subunits alpha (PSME1) and beta (PSME2) [21]. Additionally, an upregulation of some proteasomal genes has been shown in PBMCs of patients with systemic autoimmune disorders including patients with PM [22].

Based on the existing but so far indirect assumption of an involvement of the proteasome system in the pathogenesis of IIM, we investigated the expression of all catalytic proteasome subunits in inflammatory and non-inflammatory myopathies in order to search for an activation of the 20S core complex in patients with autoimmune myositis. Results were validated and comprehensively screened in a large panel of disease related as well as cell type and stimulation specific transcriptome data sets in order to confirm our results and to identify induction mechanisms and the regulatory network for the immune proteasome subunits.

## Materials and Methods

### Ethics Statement

The study was performed in accordance with the 1964 Declaration of Helsinki and approved by the “Charité University Medicine ethics committee I of Charité Campus Mitte” and patients provided written consent to participate in the study.

### Patients and healthy donors

Expression of proteasome subunits was investigated in 17 patients with idiopathic inflammatory myopathies (IIM), including polymyositis (PM, n = 5), dermatomyositis (DM, n = 5), and overlap-syndromes with myositis (OM, n = 7) (8 male, 9 female, mean age 54.3 years, age range 22–72 years). Patients were selected based on typical clinical symptoms, laboratory and/or muscle biopsy findings, which were indicative for the different groups of myopathies. Patients with PM and DM fulfilled the classification criteria according to Bohan and Peter [23], [24]. For details of diagnosis and clinical parameters see supplementary table S1. Autoantibody screening was performed according to diagnostic standards including ANA in indirect immunofluorescence on HEp2 cells. Depending on the result of the ANA pattern, further differentiation of the antibody reactivity was performed for detection of ENA using ELISA. Furthermore, a profile of myositis specific autoantibodies including anti-Jo1-, anti-SRP-, anti-Mi2, anti-PM/Scl and anti-U1RNPantibodies as well as anti-proteasomal antibodies was analyzed in each suspected case of myositis. Controls included 7 patients with different non-inflammatory myopathies (NIM, 4 male, 3 female, mean age 48.7 years, age range 35–59 years). Time and patient matched samples of muscle biopsies and blood were taken from 14 patients. Control samples from 15 healthy donors (HD, 2 male, 13 female, mean age 45 years, age range 27–56 years without clinical signs of disease, no clinical signs of muscular weakness, no medication) included only blood and no muscle biopsies. All patients were diagnosed at the Department of Rheumatology and Clinical Immunology, Charité – University Medicine Berlin and informed consent was obtained from all subjects.

### Isolation of peripheral blood mononuclear cells (PBMCs) and cellular subsets

PBMCs were collected by Ficoll density gradient centrifugation (Biochrom, Germany) and divided into two fractions. One was used for separation of dendritic cells (DCs) by Magnetic Cell Separation (MACS) using the Blood Dendritic Cell Isolation Kit-II (Miltenyi Biotec, Germany), the other for isolation of T lymphocytes (CD4+, CD8+), B lymphocytes (CD19+), and monocytes (CD14+) by fluorescence activated cell sorting (FACS) with a FACS DiVa Flow Cytometer (BD, Germany).

### Collection of muscle biopsies

Muscle tissues were stabilized in RNA later (Qiagen, Germany) to avoid RNA degradation and were stored at −70°C. For RNA isolation, biopsies were ground with a pestle and mortar in the presence of liquid N_2_ to protect RNA against degradation. Disrupted samples were then added to lysis buffer containing β-mercaptoethanol.

### RNA isolation, reverse transcription into cDNA and real-time reverse transcriptase-polymerase chain reaction (real time RT-PCR)

RNA was isolated from muscle tissues and blood cells using the NucleoSpin RNA/Protein kit (Macherey-Nagel, Germany). First-strand cDNA was synthesized using the SuperScript III First-Strand Synthesis System for RT-PCR (Invitrogen, Germany) according to the instructions of the manufacturer.

Forward and reverse primers of proteasome subunits PSMB5, PSMB6, PSMB7, PSMB8, PSMB9 and PSMB10 for real time RT-PCR were designed as described elsewhere [25].

Amplification reactions contained SYBR Green PCR Master Mix (Applied Biosystems), 200 nM forward and reverse primers for each gene and cDNA. Real-time PCR was performed in triplicates using the ABI prism 5700 Sequence Detection System (Applied Biosystems).

**Figure 1:**
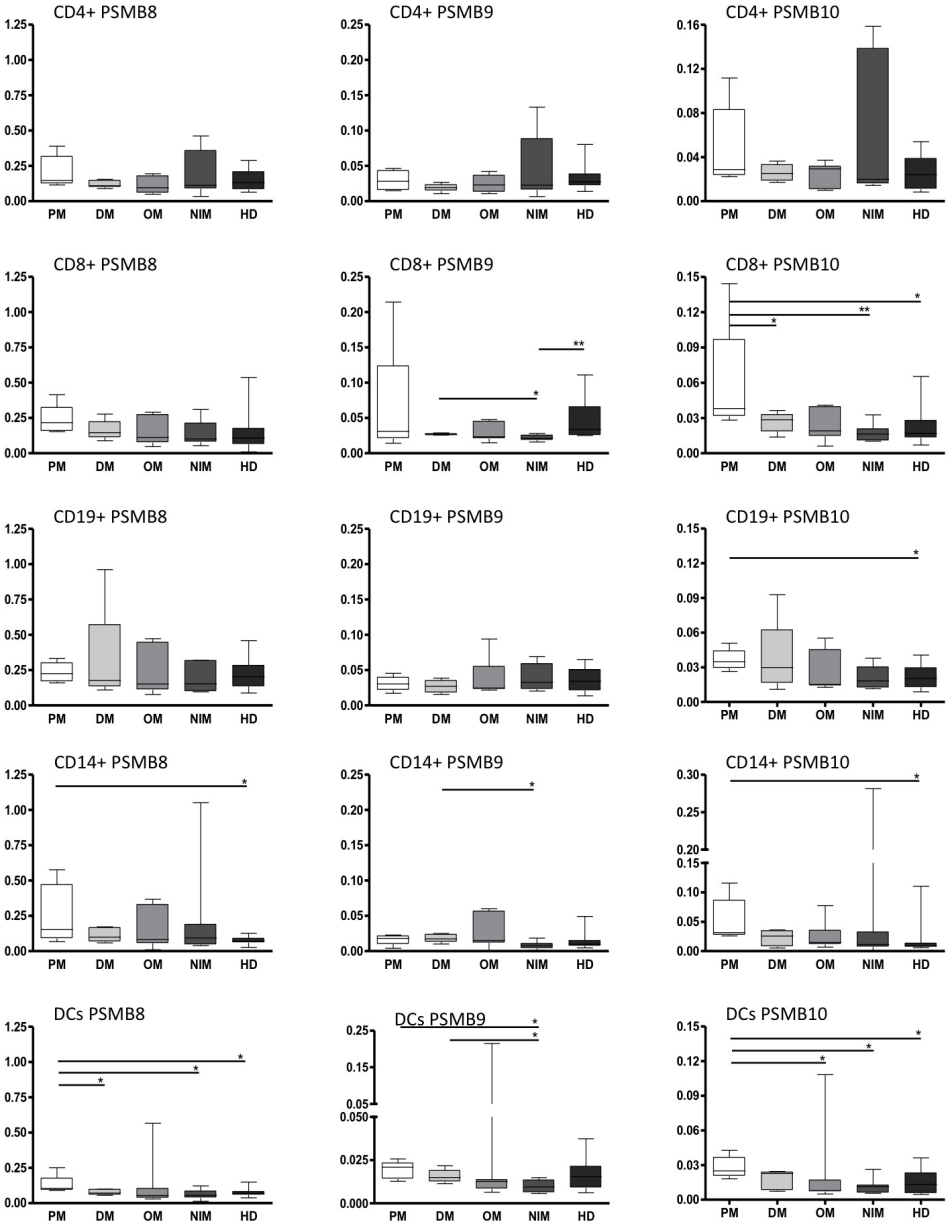
Expression of immunoproteasomal subunits in immune cells: Gene expression of immunoproteasomal subunits (PSMB8–10) in CD4+, CD8+, CD19+, CD14+ and DCs of patients with myopathies (PM, DM, OM, NIM) and controls (HD). Data are shown as relative expression normalized to beta actin. Box plots indicate percentiles 0, 25, 50, 75 and 100. Groups were compared by Mann-Whitney U test and statistical significance is indicated for p<0.05 (*) and p<0.01 (**). Significantly higher expression of PSMB8 was observed in CD14+ cells and DC of PM patients compared to HD or DM, NIM and HDs, respectively. PSMB9 was increased in CD8+ and CD14+ of DM and in DCs of PM and DM patients compared to NIM. PSMB10 was found increased in PM patients compared to DM, NIM and HD in CD8+, compared to HD in CD19+ and CD14+ cells and compared to OM, NIM, and HD in DCs.

**Figure 2:**
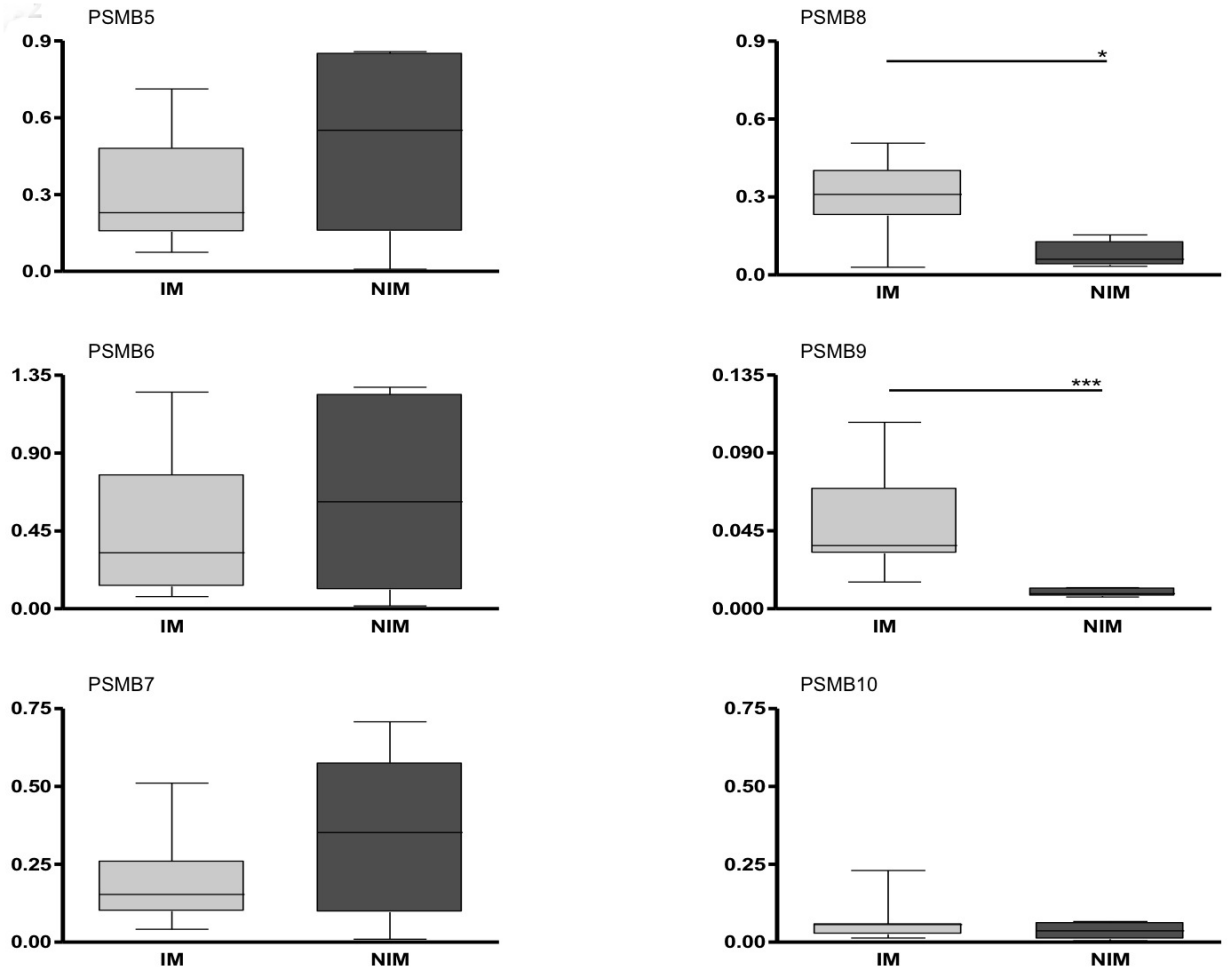
Expression of catalytic proteasomal subunits in muscle: Gene expression analysis of constitutive and immunoproteasomal subunits (PSMB5–10) in muscle biopsies of patients with inflammatory myopathies (IM) and patients with non inflammatory myopathies (NIM). Data are shown as relative expression normalized to beta actin. Box plots indicate percentiles 0, 25, 50, 75 and 100. Groups were compared by Mann-Whitney U test and statistical significance is indicated for p<0.05 (*), p<0.01 (**) and p<0.001 (***). Comparing to NIM, mean relative expression levels in IM revealed 4-fold for PSMB8 [0.302±0.139 and 0.075±0.041] and about 5-fold increase for PSMB9 [0.049±0.029 and 0.009±0.002] but less than 2-fold for PSMB10 [0.065±0.064 and 0.036±0.022].

**Figure 3:**
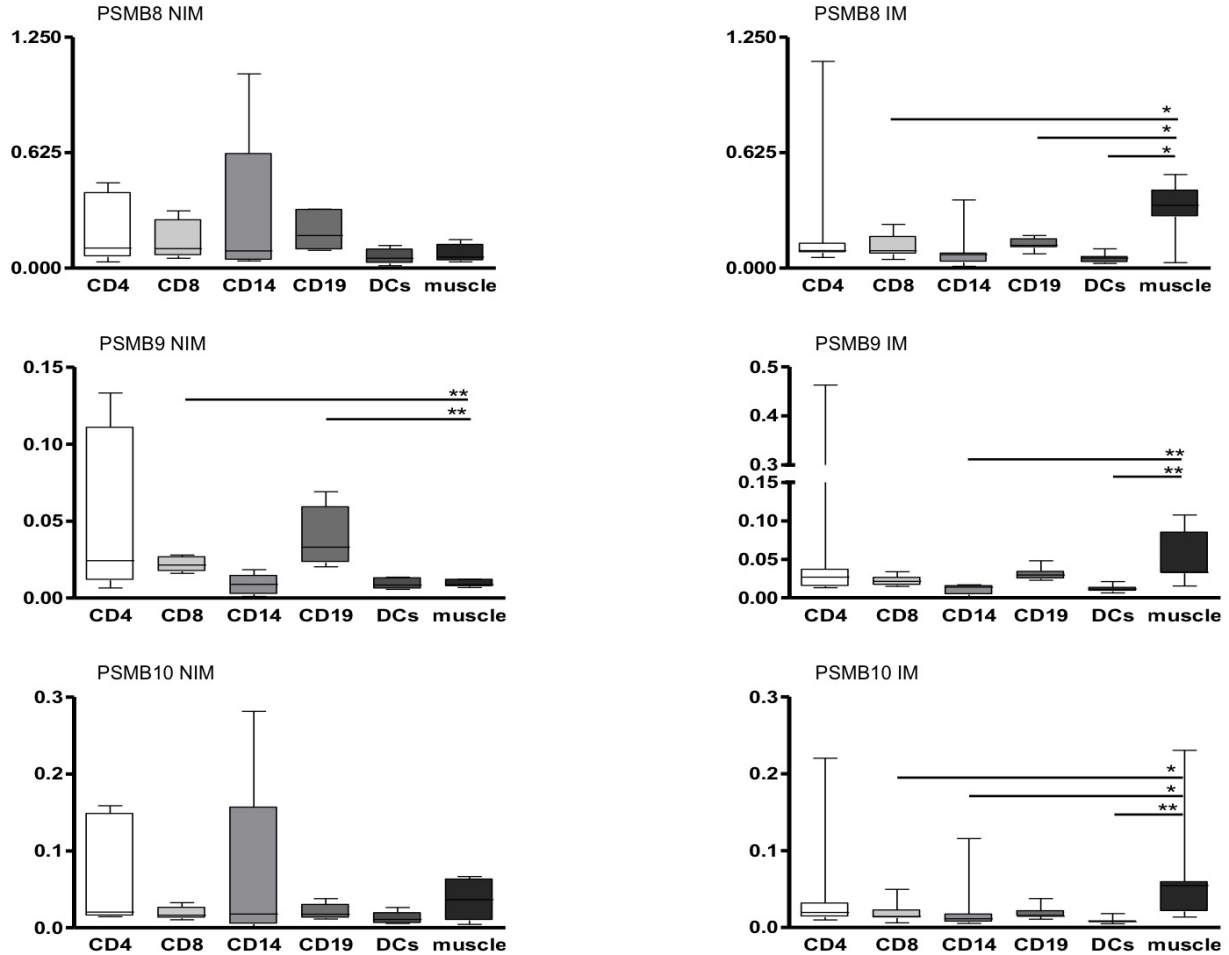
Comparison of immunoproteosomal subunit expression between paired samples from isolated cells and muscle tissue: Gene expression analysis of immunoproteasomal subunits (PSMB8–10) in muscle biopsies vs. CD4+, CD8+, CD14+, CD19+, and DCs from patients with inflammatory myopathies (IM) and patients with non-inflammatory myopathies (NIM). Data are shown as relative expression normalized to beta actin. Box plots indicate percentiles 0, 25, 50, 75 and 100. Groups were compared by Mann-Whitney U test and statistical significance is indicated for p<0.05 (*) and p<0.01 (**).

Relative expression of the target compared to the house keeping gene beta-actin was determined as 
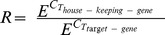
 [26], with E representing the amplification efficiency of the respective primer system. Mann-Whitney U-test was applied for group comparisons (figures 1, 2, 3).

### Western blot analysis

**Figure 4:**
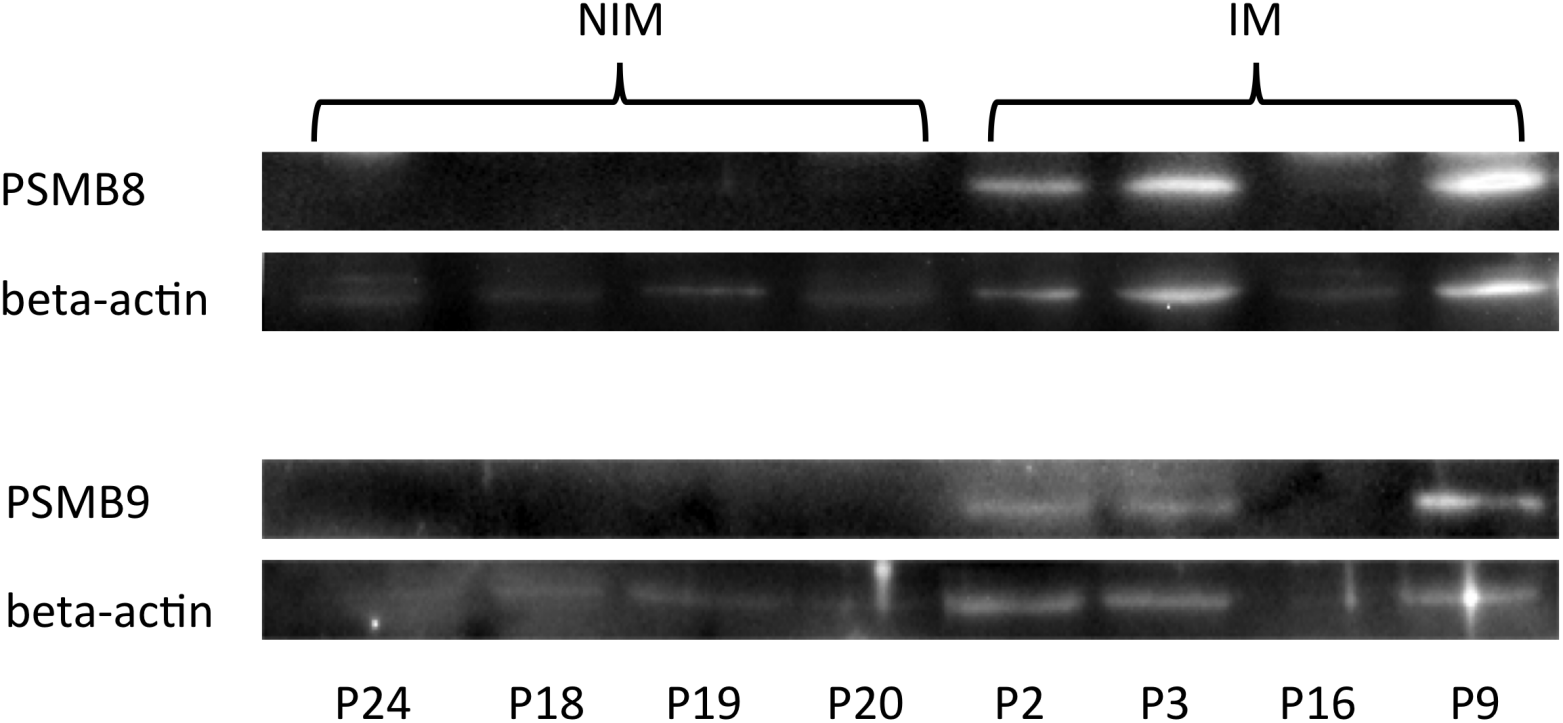
Protein expression of the immunoproteasome subunits PSMB8 and PSMB9 relative to beta actin in IM and NIM. The protein expression of the two subunits was analyzed by western blot in muscle biopsies from four non-inflammatory myopathies (NIM) and four inflammatory myopathies (IM). Intensity of chemiluminescent signals for PSMB8 and PSMB9 was normalized to corresponding signals of the housekeeping protein beta actin, which was detected in a second staining procedure on the same membrane. Only IM samples revealed PSMB8 and -9 protein expression.

Muscle biopsies were minced in the presence of radioimmunoprecipitation assay (RIPA) lysis buffer. PBMCs were used as positive control and treated with the same lysis buffer. 30 µg of total protein from tissue homogenate or cell lysate were loaded and fractionated by 15% sodium dodecyl sulfate polyacrylamide gel electrophoresis. After transfer to a polyvinylidene fluoride (PVDF) membrane, the blots were incubated overnight at 4°C with monoclonal antibodies against proteasome PSMB8 or proteasome PSMB9, both diluted 1∶1000 (Enzo Life Sciences, USA). Staining was performed with polyclonal rabbit anti mouse immunoglobulins conjugated with horseradish peroxidase (1∶1000; Dako, Denmark) and visualised by enhanced electrochemiluminescence Pierce ECL Western Blotting Substrate (Thermo Scientific, USA) (figure 4). For quantification relative to a housekeeping gene, beta actin was detected using the same procedure and mouse anti-beta actin diluted 1∶10,000 as primary antibody (Sigma, USA). Quantification on western blot images was performed with the intensity histogram function of Adobe Photoshop (Munich, Germany).

### Microarray data and statistical analysis

For validation, transcriptome data of muscle biopsies were collected from the open access database Gene Expression Omnibus (GEO, http://www.ncbi.nlm.nih.gov/geo/). The selection included muscle tissue biopsies from different types of IIM, non-inflammatory myopathies, septic patients, volunteers after IL-6 infusion and healthy controls as well as purified dendritic cells from peripheral blood (GSE2044, GSE3112, GSE5370, GSE39454, GSE3307, GSE13205, GSE10685 and GSE23618; table S2). Own data included transcriptomes of stimulated monocytes (GSE38351) [27] and sorted immune cell populations (CD14+ monocytes, CD15+ granulocytes, CD4+ T-cells, CD8+ T-cells, CD19+ B-cells and CD56+ NK-cells, each n = 3; GSE58173) from peripheral blood of healthy donors, generated after RNA extraction, amplification and hybridization to Affymetrix GeneChip HG-U133 Plus 2.0 arrays according to standard protocols to minimize influence on gene expression by sorting [28]. All data were analysed in the BioRetis database (www.bioretis.de) using standard algorithms [29]. Selection of differentially expressed genes was performed by “default increased” filtering in BioRetis [29]. Probesets were ranked by an equally weighted sum-score for “SLR” and “frequency of increase” in disease compared to control (table S3).

**Figure 5:**
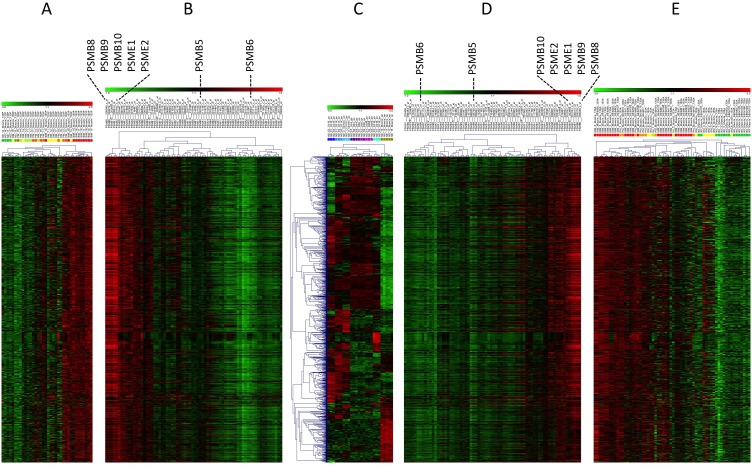
Correlation of proteasome subunit expression with myositis signatures and overlap with immune cell infiltration: Expression of all proteasome units were investigated in transcriptome data of muscle biopsies referenced in table S2 and were correlated with genes increased in myositis muscle tissue. The heatmap of the probeset signal intensities (A) is opposed to the heatmap of the gene by gene correlation matrix (B) in 133P array experiments. Identical analysis in 133A arrays are shown in D and E. Expression of the 1209 probesets in reference transcriptomes of purified immune cells from the blood of healthy donors served as basis for clustering by probesets and cell types (C). Identification of cell type specific transcriptional patterns in the 1209 probesets demonstrates the extent of overlap of these probesets with immune cell related transcripts. Myositis samples include IBM (red), PM (orange), DM (yellow), NM (necrotizing myopathy)/IM (inflammatory myopathy) (light green) and HD (green). Immune cell types include neutrophils (dark blue), monocytes (blue), dendritic cells (light blue), CD4+ T-cells (dark violet), CD8+ T-cells (violet), NK-cells (light violet), B-cells (cyan), and healthy muscle (moss-green). A detailed graph with all probesets labelled is presented in supplemental material (figure S4).

**Figure 6:**
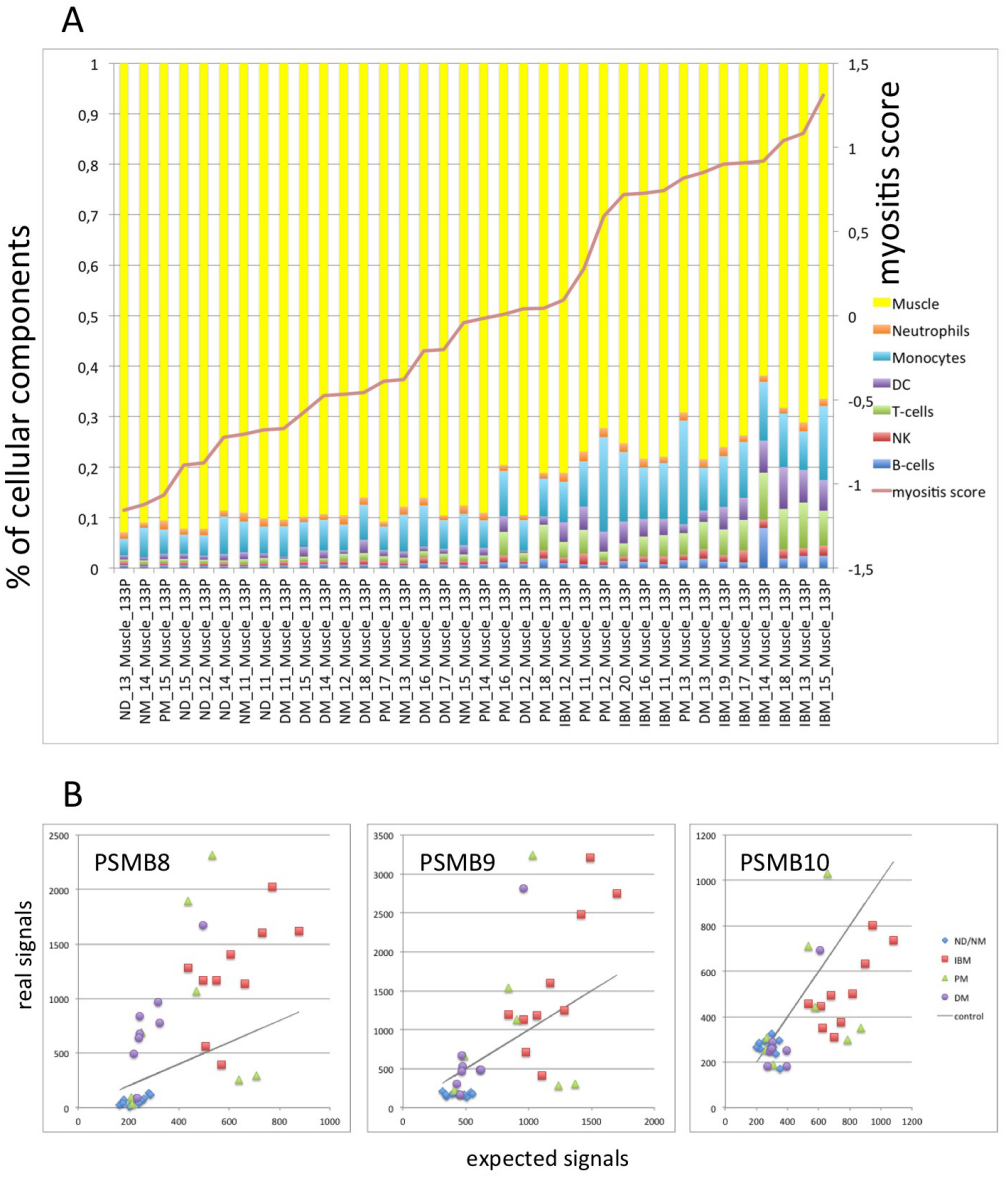
Quantification of cellular infiltration and confirmation of PSMB8/-9 gene activation in myositis muscle samples: Transcriptome data referenced in table S2 were re-investigated. A) Cell type infiltration was quantified based on the expression of cell specific marker transcripts (figure S5). Signal ratios between muscle biopsy and purified cell type were calculated for each cell type specific transcriptional markers. Taking the median of the ratios in each cell specific marker set and scaling their sum to 100% revealed an estimate of the cellular composition. Increase of monocyte, dendritic cell and T-cell transcription patterns corresponds to the molecular myositis score. (ND = normal donor; NM = necrotizing myopathy). B) The maximum of infiltration related signal intensity was calculated as described in materials and methods. Comparing these expected intensities (x-axis) with real intensities (y-axis) in each sample, PSMB8 and PSMB9 are higher expressed than expected in the majority of IBM and several of the PM muscle. In DM, PSMB8 expression also exceeds the expected signal intensity, although on a lower level. One out of the 8 DM samples (DM_13) showed consistently a pattern similar to the highly inflamed IBM samples. All other conditions including controls remained below the expected intensity. For PSMB10, the increased signal intensity in several samples of IBM seems to correspond to the level of infiltration.

Signals were quantile normalized for evaluation of cell infiltration and correlation analysis (figure 5). Percentage of immune cell infiltration was estimated using tissue and cell type specific marker probesets by comparing the reference transcriptomes of each individual cell type and healthy muscle tissue with each other (figure 6). To assess the maximum of infiltration related signal intensity for the immunoproteasome transcripts in each myositis biopsy a linear model of relationship between signal intensity and percentage of infiltration was applied. The highest expected signal (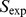) related to infiltration was calculated as 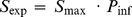 with 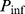 as the percentage of all non-muscle cell types (infiltrated cells) and 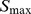 as the maximum signal observed in the reference transcriptomes of any of the purified immune cells from healthy donor. The median of log-transformed and z-normalized signals of all 1209 myositis related probesets was used for scoring and sorting of the samples (myositis score).

## Results

### Dendritic and monocytic cells of the peripheral blood are the leading cell type for immunoproteasome upregulation in myositis

Relative quantification of proteasomal subunit expression by real time RT-PCR revealed a myositis related significant increase of at least one of the immunoproteasomal subunits PSMB8, PSMB9 and/or PSMB10 in all investigated cellular subsets except CD4+ (figure 1). DCs were the leading subset with differences in all immunoproteasomal subunits followed by monocytes, CD8+ and finally CD19+ lymphocytes. Differences were most prominent in PM and to a lower extent in DM patients when compared to non-inflammatory myopathies (NIM) and healthy donors (HD). Differences between immunoproteasomal and corresponding constitutive subunits were especially found between PSMB8 and PSMB5 in all investigated peripheral blood cell types of all patients (figure S1) and HD (data not shown).

### Immunoproteasomal subunit expression is higher in IIM than NIM muscle biopsies and indicates a substitution of constitutive subunits

In all patients with inflammatory myopathies (IM) including PM, DM and OM, immunoproteasomal subunits PSMB8 and PSMB9 were significantly increased when compared to NIM patients (figure 2). In contrast, PSMB10 was less increased in IM patients compared to NIM and not statistically significant. The ratios between immunoproteasomal and corresponding constitutive subunits (PSMB8/PSMB5, PSMB9/PSMB6 and PSMB10/PSMB7) in IM [1.79, 0.35 and 0.42, respectively] compared to non-inflammatory biopsies [0.94, 0.1 and 0.18, respectively] indicate an imbalance and suggest substitution of constitutive by immunoproteasomal subunits in patients with IIM.

### Immunoproteasomal subunit expression in IIM is higher in muscle compared to donor matched immune cells of peripheral blood

In paired samples of blood and tissue from 7 IM and 6 NIM patients collected at the same time, real time RT-PCR revealed significant increase of immunoproteasomal subunits in muscle compared to blood cells only in IM. In contrast, in NIM, expression of immunoproteasomal subunits was higher in most blood cell types compared to muscle tissue (figure 3). Constitutive subunits were higher expressed in all muscle biopsies from IM and NIM when compared to any cell type of the blood (data not shown).

### Regulation of PSMB8 and PSMB9 protein in myopathy patients

To confirm that the elevated transcriptional activity is also translated into protein, PSMB8 and PSMB9 proteins were investigated in muscle tissue of representative patients with dermatomyositis (P2 and P3), polymyositis (P9), overlap syndrom (P16) and non-inflammatory muscle diseases (P18, P19, P20, P24). Western blot analysis revealed equal or higher protein detection intensities of PSMB8 compared to actin in the patients with inflammatory muscle diseases and slightly weaker intensities for PSMB9. In all samples investigated from non-inflammatory diseases both immunoproteasomal subunits were not visible as a distinct band (figure 4). Intensities determined for PSMB8/-9 relative to actin were also significantly lower in NIM than in IIM. Correlation between immunoproteasomal protein and RNA transcript expression, both as relative quantities compared to actin protein or transcript, revealed high correlation coefficients of R = 0.90 for PSMB8 and R = 0.80 for PSMB9.

### Transcriptome data for validation and pathophysiologic classification of defined transcripts

To validate these results and to characterize the importance of immunoproteasomes in myositis compared to other molecular mechanisms, we investigated open access transcriptome data of muscle biopsies from patients with IIM and NIM as well as from healthy donors. Transcriptomes from different types of immune cells and stimulation experiments with IFN, TNF and LPS were selected to address functional interpretation (table S2).

#### Immunoproteasomal subunits PSMB8/-9 are leading candidates in the pathophysiology of IIM muscle inflammation

Muscle transcriptomes from patients with IBM, PM and DM were compared to healthy controls and revealed 1209 probesets equal to 927 genes, which were upregulated in at least one of the myositis diseases (table S3) as a molecular correlate of disease activity. PSMB8 ranked at position 9 and PSMB9 at position 58 out of the 927 genes when scored for magnitude and consistency of increase. Based on these 1209 probesets, IIM samples did not cluster by disease specific patterns but overall intensity of molecular changes. High molecular activity was most frequent in IBM followed by PM and DM muscle biopsies. This was observed independently for both 133A and 133P data sets (figure 5A, 5E, S2). Of all known proteasomal subunits, PSMB8/-9 as well as their activator subunits PSME1 and -2 [30] correlated best with the 1209 probesets while most of the constitutively expressed subunits including PSMB5 and -6 were inversely correlated. This was again independently observed in 133A and 133P datasets (figure 5B and 5D). Characterizing the panel of 1209 probesets with DAVID (http://david.abcc.ncifcrf.gov/) revealed that almost each gene annotated to the antigen processing and presentation pathways of MHC-I and II was included (figure S3).

#### Transcription of PSMB8/-9 is actively up-regulated in IIM muscle tissue

Investigating gene expression for the 1209 probesets in reference signatures of healthy muscle and immune cell populations uncovers that the majority is also part of the physiologic expression in immune cells and is sufficiently specific to correctly cluster the immune cell profiles (figure 5 C tree of clustered samples). Thus, these transcripts could be unregulated but passively transported by cell infiltration into muscle or also upregulated by additional gene activation (figure 5). Such active upregulation seems to occur for transcripts specific for healthy muscle (bottom of figure 5 C), which were increased in myositis. A magnification of figure 5 with detailed description of genes is provided in figure S4.

Compared to immune cells, there is no relevant expression of immunoproteasomes in healthy muscle. Selection of optimized cell type specific marker transcripts (figure S5) disclosed quantitatively the cell type specific transcriptional activities in the muscle biopsies, which were besides muscle especially related to T-cells, monocytes and dendritic cells and corresponded to the overall molecular change in myositis muscle transcriptomes (myositis score; figure 6 A). Comparing real expression levels of immunoproteasome subunits in myositis with intensities expected from immune cell infiltration demonstrated that PSMB8/-9 were actively upregulated in patients with IBM, PM and DM, especially when high expression levels not only of the immunoproteasomal subunits but also of genes differentially expressed in inflammatory myopathies compared to healthy muscle were observed and thus were indicative for high molecular disease activity (figure 6 B). PSMB10 expression, however, matched with expected intensities, which indicated no additional upregulation.

#### Transcription factors STAT1 and IRF1 correlate with upregulation of PSMB-8 and -9 expression in IIM and in IFN stimulated monocytes

Regulatory mechanisms for immunoproteasomes in myositis were searched by correlation of transcription factors defined by GO:0003700 with PSMB8/-9 in all muscle biopsy samples. This revealed STAT1, IRF1, TRIM22 and IRF9 as potential regulators, which were predominantly increased in IBM, but also present in many PM and several DM samples. PSMB5/-6 were not or inversely correlated. In different healthy donor immune cell types and healthy muscle, correlation between PSMB8/-9 and these transcription factors was much weaker. However, monocytes stimulated with TNF, LPS, IFNα2 or IFNγ revealed highest correlation between PSMB8/-9 and STAT1 followed by IRF1 (table S4). This was related to IFNα2 and IFNγ. In contrast, TNF suppressed both transcription factors and PSMB8/-9, while LPS induced both transcription factors but suppressed PSMB8/-9 expression (table S4).

#### In IIM, IFNγ but not type-1 IFN correlate with PSMB8/-9 expression and T-cell markers including CD8

**Figure 7:**
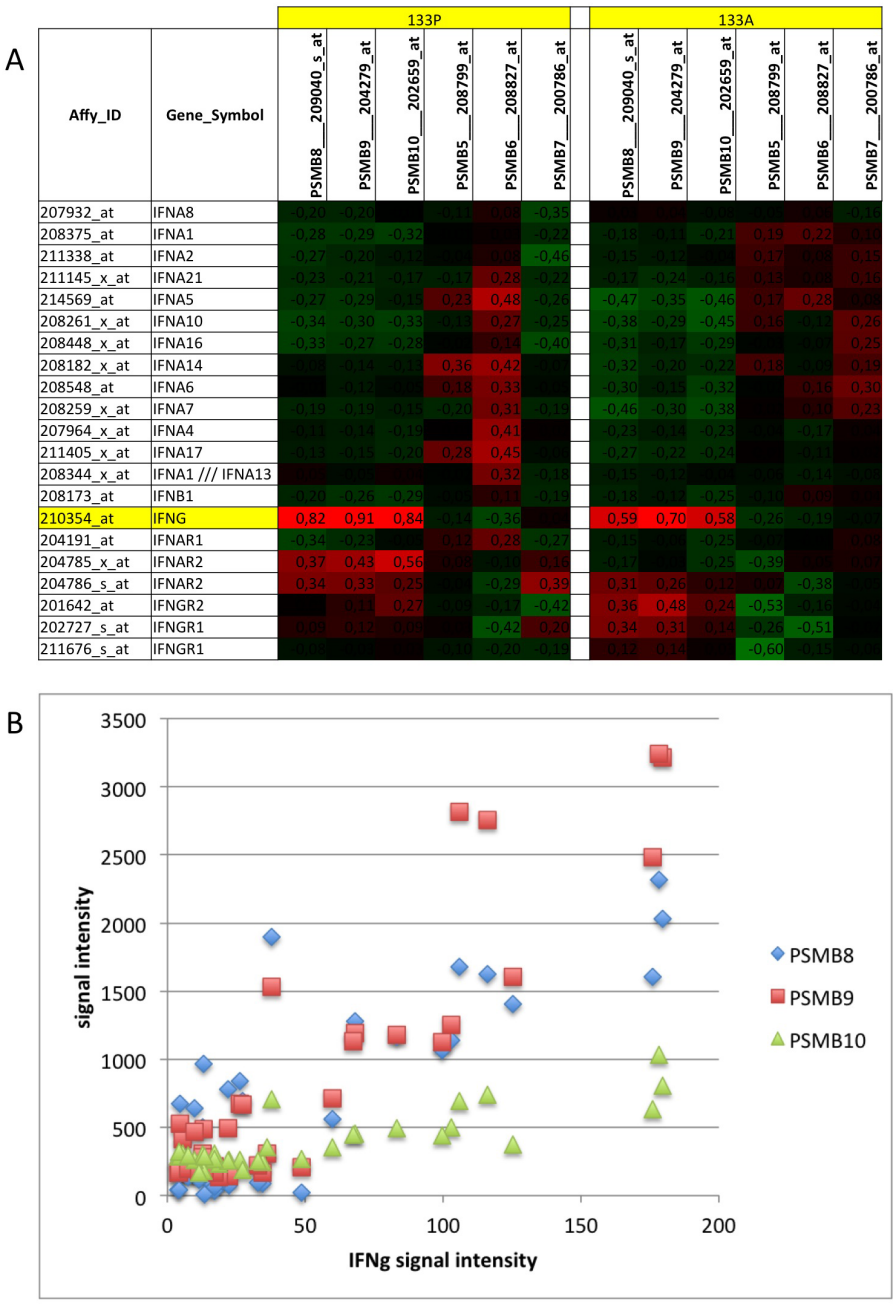
Correlation of immunoproteasome with IFN and IFNR expression in myositis muscle tissue: Transcriptome data referenced in table S2 were re-investigated. A) Correlation analysis was performed for 133P and 133A datasets independently. Only IFNγ revealed high correlation coefficients with PSMB8, PSMB9 and PSMB10. The corresponding constitutively expressed subunits PSMB5-7 were not or even negatively correlated. B) Comparing the association of IFNγ with PSMB8, -9 and -10 for each sample individually, the increase was much higher for PSMB8/-9 compared to PSMB10 as described before (figure 6).

Although both types of interferons may induce PSMB8/-9 [27], in myositis muscle transcriptomes only IFNγ was significantly increased with dominance in IBM (74% increased change call, 4.9-fold increased) and correlated with PSMB8/-9. These analyses were separately performed with 133P and 133A data sets and independently confirmed each other (figure 7). To trace the cellular origin, signals of IFNγ in myositis were correlated with all cell type specific marker transcripts and the median of all correlation coefficients per cell type was calculated. IFNγ expression correlated best with T-cells (133P: R = 0.78; 133A: R = 0.62) and CD8 (133P: R = 0.80; 133A: R = 0.69) followed by dendritic cells (133P: R = 0.66; 133P: R = 0.57) in both, 133A and 133P datasets. NK-cell association was also high in 133P (R = 0.54) but much lower in 133A datasets (R = 0.17).

#### Immunoproteasome activation is specific for IIM but not non-inflammatory myopathies

Reanalysis of muscle transcriptomes from 12 different muscle diseases (GSE3307) with non-inflammatory myopathies except from juvenile dermatomyositis (JDM) revealed upregulation of PSMB8/-9 only in samples from JDM and in one out of four samples from limb girdle muscular dystrophy 2I (LGMD2I). Furthermore, muscle biopsies from healthy donors after IL-6 infusion (GSE10685) or patients with sepsis induced multiple organ failure (GSE13205) did not reveal any increase of the immunoproteasome expression, indicating that expression of PSMB8/-9 in muscle is specific for IIM.

## Discussion

With this first comprehensive analysis of muscle biopsies and blood derived immune cells in myositis on the catalytic subunits of the proteasome, we could demonstrate a central role of immunoproteasomes in the inflammatory process of IIM including IBM, PM and DM on transcriptional as well as protein expression level. We identified PSMB8/-9 upregulation only in combination with infiltration of antigen presenting cells and associated with the expression of IFNγ and CD8 accompanied by T-cell infiltration in biopsies of inflamed muscle. This perfectly integrates into current concepts of IIM pathophysiology and extends these towards a strong involvement of mechanisms and modulations of antigen processing.

IIM are of unknown aetiology and present with characteristic upregulation of MHC-class I and II molecules [1], [31], which was also detectable in the myositis transcriptome data. Antigen presentation is affected by replacement of constitutive with immunoproteasomal subunits [13]. In this study, we observed that physiologic expression of the immunoproteasome subunits in healthy controls was not only restricted to professional antigen presenting cells but found in all subtypes of peripheral blood immune cells. Healthy muscle tissue in contrast expressed only constitutive subunits while immunoproteasome expression was negligible. Immunoproteasomal processing is especially linked to the MHC-I pathway [13]. Thus, it is important that in our investigation transcription was highly correlated with protein production of immunopreteasomes and also associated with the histological finding of MHC-I protein upregulation in muscle tissue of the analyzed IIM samples.

Our reanalysis of the myositis transcriptomes revealed that molecular changes of active inflammation were mainly associated with an increase of immune cell transcriptome signatures and interestingly independent from clinical diagnosis. Immunoproteasome subunit expression in IIM was upregulated especially in DC and monocytes of the peripheral blood but also in muscle, where PSMB8/-9 correlated with the molecular changes of inflammation and thus infiltration of immune cells. The increase of PSMB8/-9 expression in IIM was higher than the level expected by infiltration of immune cells and was also higher in total biopsy material with an immune cells fraction below 40% compared to any of the purified (100%) donor matched immune cells from peripheral blood. This clearly indicates that these immunoproteasomal transcripts must be upregulated in one or more of the cell types of inflamed muscle.

Recently, transcriptomes obtained from type I and II IFN stimulated monocytes [27] revealed similar expression patterns of immunoproteasomal subunits with upregulation of PSMB8/-9 but not -10, suggesting the involvement of type I and/or type II IFN triggers on PSMB8/-9 regulation in inflamed muscle tissues. IFNγ was reported to induce MHC-II and enhance MHC-I expression in affected muscles of PM and DM patients [32]. Recently, an IFNα signature and influence is discussed especially in DM but also in PM [33]. In contrast, a role of IFNγ triggering is assumed especially in IBM [34]. In this study, we could identify in the myositis transcriptomes only IFNγ but not IFNα as a predominant trigger for PSMB8/-9, which also correlated with the expression of STAT1 and IRF-1. Both transcription factors were also reported to mediate IFNγ induced PSMB9 expression in murine macrophages [35]. Interestingly, IFNγ production by T-cells was reported to depend on immunoproteasomes [36]. Especially in IBM the link of MHC-I with immunoproteasomal units suggests an important role for antigen processing via PSMB8 and -9 dependent mechanisms. This may be induced by misfolded protein from fiber degradation or suspected retroviral or viral triggers in this type of myositis [37] and thereby contribute to CD8+ T-cell triggering, expansion and IFNγ production.

Thus, immunoproteasomes and antigen processing seem to be pivotal in molecular pathomechanisms of myositis and may serve particularly as biomarker of myositis activity. This is further supported by the lack of immunoproteasome activation in our reanalysis of muscle transcriptomes from non-inflammatory myopathies [38], patients with severe inflammatory syndroms like sepsis [39] or conditions related to high IL-6 levels in the circulation [40].

This important association of immunoproteasome upregulation with myositis activity raises the discussion, whether these diseases qualify as a model for therapeutic targeting of immunoproteasomes. Inhibitors are currently developed and tested with controversial effects [41], [42]. Application of the PSMB8 selective inhibitor PR-957 in experimental arthritis or colitis could reduce cytokine production and attenuate disease activity [43], [44]. On the other hand, the recently described mutations c.224C>T (p.Thr75Met) [15], G201V [16], G197V [17] and c.405C>A [18] in PSMB8 were all associated with decreased subunit activity and different inflammatory syndroms, suggesting that immunoproteasome suppression may cause additional effects depending on dosage and cell type involvement. Studies in PSMB8/-9 deficienct mice suggested that inflammation induced immunoproteasome expression in tissue may also prevent CD8+ T-cell mediated autoimmunity [45].

Knowing that immunoproteasomes modulate antigen processing with effects on MHC-I peptide presentation [46], intracellular protein homeostasis [47] and CD8 T-cell responses [48] especially in mixed proteasomes [49], immunoproteasome inhibition may be a double-edged sword. Influencing antigen processing and presentation may reduce CD8+ T-cell triggering through MHC-I but may also increase toxicity by accumulating misfolded proteins.

In summary, our results support the hypothesis that the proteasome system is activated and contributes to a perpetuating crosstalk between antigen-presenting cells and T-cells via immunoproteasome generated peptides and IFNγ. Assuming altered autoantigen processing as driving mechanism, suppression of the immunoproteasome could be a promising therapeutic concept. Therefore, further studies are needed that focus on antigens and peptides, which are specifically processed by immunoproteasomes in IIM as well as on mechanisms of suppressing or modulating antigen processing and presentation.

## Supporting Information

Figure S1 **High expression levels of PSMB8 compared to PSMB5 in all isolated cells:** Gene expression of constitutive (PSMB5-7) and immunoproteasomal subunits (PSMB8-10) in CD4+, CD8+, CD19+, CD14+, DCs and muscles of all patients. Data are shown as relative expression normalized to beta actin. Box plots indicate percentiles 0, 25, 50, 75 and 100.
(TIF)

Figure S2 **Differences in upregulation of myositis related genes between IBM, PM, DM, NM and IM:** All 1209 probesets were sorted by a sum-score for magnitude and frequency of increase in myositis. The heat map presents each disease group by the mean values of the signal intensity in all samples of the group. Combined scoring according to analysis on the 133A and 133P platform demonstrates that the strongest increase is observed in IBM followed by PM and DM, while IM and NM were closest to healthy control. This pattern was observed in 133A samples as well as in 133P samples independently of combined scoring (A and B) or scoring based on each individual platform (C and D).
(TIF)

Figure S3 **Identification of genes involved in MHC-I and MHC-II antigen processing and presentation pathways:** The 1209 probesets upregulated in myositis were uploaded into the DAVID database (http://david.abcc.ncifcrf.gov/) for functional annotation. All genes highlighted with a red star are included in the 1209 probesets.
(TIF)

Figure S4 **This is the corresponding image to** **figure 5** **in the manuscript.** It lists all gene names and is provided as an additional jpg-file “Figure_S4” for further magnification (http://www.charite-bioinformatik.de/supplementary_data/immunoproteasomes/04_Sj9CPykssy0xPLMnMz0vMAfGjzOLNLU_Figure_S4.jpg).
(TIF)

Figure S5 **Cell type specific transcripts and corresponding changes of gene expression in myositis:** Cell type specific transcripts were determined from transcriptomes of monocytes, neutrophils, CD1+ dendritic cells, T-cells, B-cells, NK-cells and muscle tissue by filtering for cell type specific transcripts with signal level >2000 in the population of interest, <200 in all other populations and a fold change of >20 if possible. In the heatmap on the right side, there is some overlapping expression in the different types of phagocytic cells and in the different lymphocyte populations. CD4+ and CD8+ T-cells do not allow the establishment of a transcript pattern that will distinguish them from other cell types and at the same time will differentiate between these two T-cell subpopulations. In the heatmap on the left side, all myositis transcriptomes were mapped to these marker panels and samples were sorted by intensity of change in the 1209 “myositis genes”. This was performed using the median of log-transformed and z-normalized signals of all 1209 probesets for each sample as a score (myositis score). Sorting myositis samples from the lowest score on the left side (predominantly normal donor samples) to the highest score on the right side (predominantly IBM samples), there is an increase especially of transcripts related to monocytes, dendritic cells and T-cells corresponding to the severity of myositis with a corresponding decrease of muscle specific transcripts. (Figure S5 is also provided as an additional separate jpg-file for further magnification: http://www.charite-bioinformatik.de/supplementary_data/immunoproteasomes/yMDI2dDbwsPIJdDBwNDNwCjLzDgowsDIEK_Figure_S5.jpg).
(TIF)

Table S1 **Clinical data of patients with DM, PM, OM and NIM.**
(XLS)

Table S2 **Collection of transcriptome data from the Gene Expression Omnibus repository:** These transcriptome data were used for analysis of the role of immunoproteasomes in inflammatory and non-inflammatory muscle diseases compared to other genes differentially expressed in myositis.
(XLS)

Table S3 **Probesets and genes identified as upregulated in IBM, PM and/or DM with signal intensities and molecular scores:** Datasets of GSE2044, GSE3112, and GSE39454 were used to identify molecular changes in IBM, PM and DM compared to healthy muscle biopsies. Data generated with the different platforms HG-U133A (133A) and HG-U133Plus 2.0 (133P) were analysed separately to avoid technical bias. Each disease entity was compared to healthy controls. Selection of differentially expressed probesets was based on the frequency of change in pairwise comparisons between arrays from two different groups, on signal log ratio (SLR), on t-test statistics and on cut-off for absolute signal intensities combined to a default filtering as provided in BioRetis. Probesets, which were upregulated in the same disease in both platforms, were selected and combined from all diseases to a total of 1209 probesets/927 genes. To score these probesets by dominance of increase, the frequency of change call for all pairwise comparisons and the SLR were z-normalized across all selected probesets and then scaled to the maximum of “1”. The sum of both normalized values was used for ranking, thus identifying genes with the best sum-score for “highly increased” and “most frequently increased” in disease compared to control in the top ranks. These probesets were sorted by a sum-score for magnitude and frequency of increase. See file “Table_S3.xls” accessible via: http://www.charite-bioinformatik.de/supplementary_data/immunoproteasomes/Tcx8DAP8zJzMDIKSjY3M_R2cDA3YCQ_nD9_Table_S3.xls. 1. sheet “probesets” with the list of the 1209 probesets sorted by the composed score of SLR and change call. 2. sheet “genes” with the list of the 927 genes sorted by the composed score of SLR and change call. 3. sheet “PSM 133P” with signals for all proteasomal units of the 133P arrays. 4. sheet “PSM 133A” with signals for all proteasomal units of the 133A arrays. Data of all microarrys and group comparisons are available in BioRetis (www.bioretis.com).
(XLS)

Table S4 **Correlation of PSMB8 and PSMB9 expression in myositis with transcriptions factors:** Correlation coefficients on the basis of signal values from myositis muscle biopsies (columns A1–A4), immune cells (B1–B2) and cytokine stimulated monocytes (C1–C2) are presented in combination with the z-normalized signal values of purified immune cells (columns D1–D21), healthy muscle profiles (E1–E5), cytokine stimulated monocytes (F1–F22), muscle biopsies based on 133P arrays (G1–G36) and 133A arrays (H1–H62); (see file Table_S4.xls download link: http://www.charite-bioinformatik.de/supplementary_data/immunoproteasomes/KFQlWFwAVoDPCrACAxzA0UDfzyM_N1W_ID_Table_S4.xls).
(XLS)
