# Supplementary figures and images for: Upregulation of Immunoproteasome Subunits in Myositis Indicates Active Inflammation with Involvement of Antigen Presenting Cells, CD8 T-Cells and IFNγ
Source: PLoS One. 2014 Aug 6;9(8):e104048. doi: 10.1371/journal.pone.0104048 (PMC4123911; doi:10.1371/journal.pone.0104048)

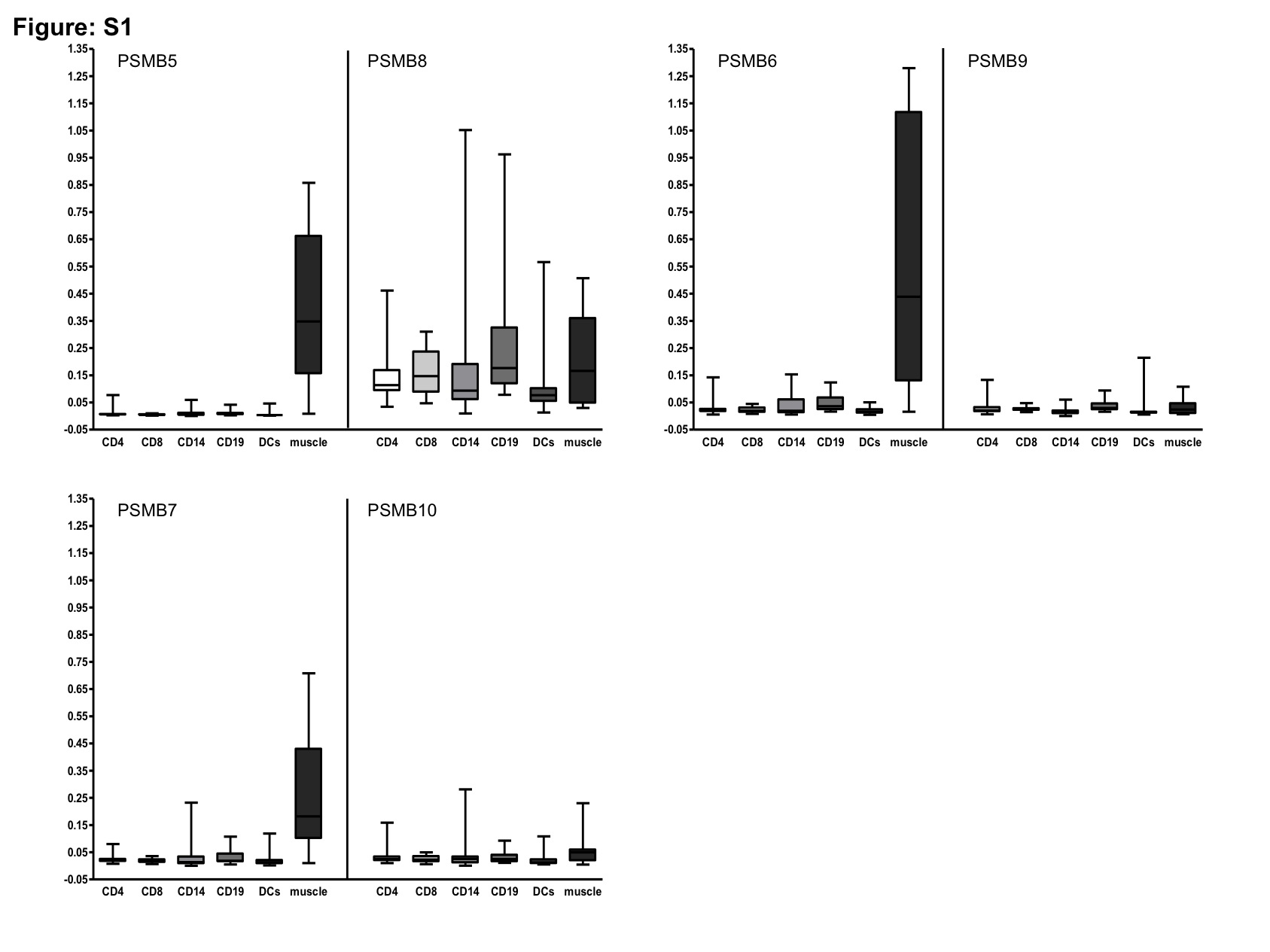

Supplement: Figure S1 — High expression levels of PSMB8 compared to PSMB5 in all isolated cells: Gene expression of constitutive (PSMB5-7) and immunoproteasomal subunits (PSMB8-10) in CD4+, CD8+, CD19+, CD14+, DCs and muscles of all patients. Data are shown as relative expression normalized to beta actin. Box plots indicate percentiles 0, 25, 50, 75 and 100. (TIF) [file pone.0104048.s001.tif]

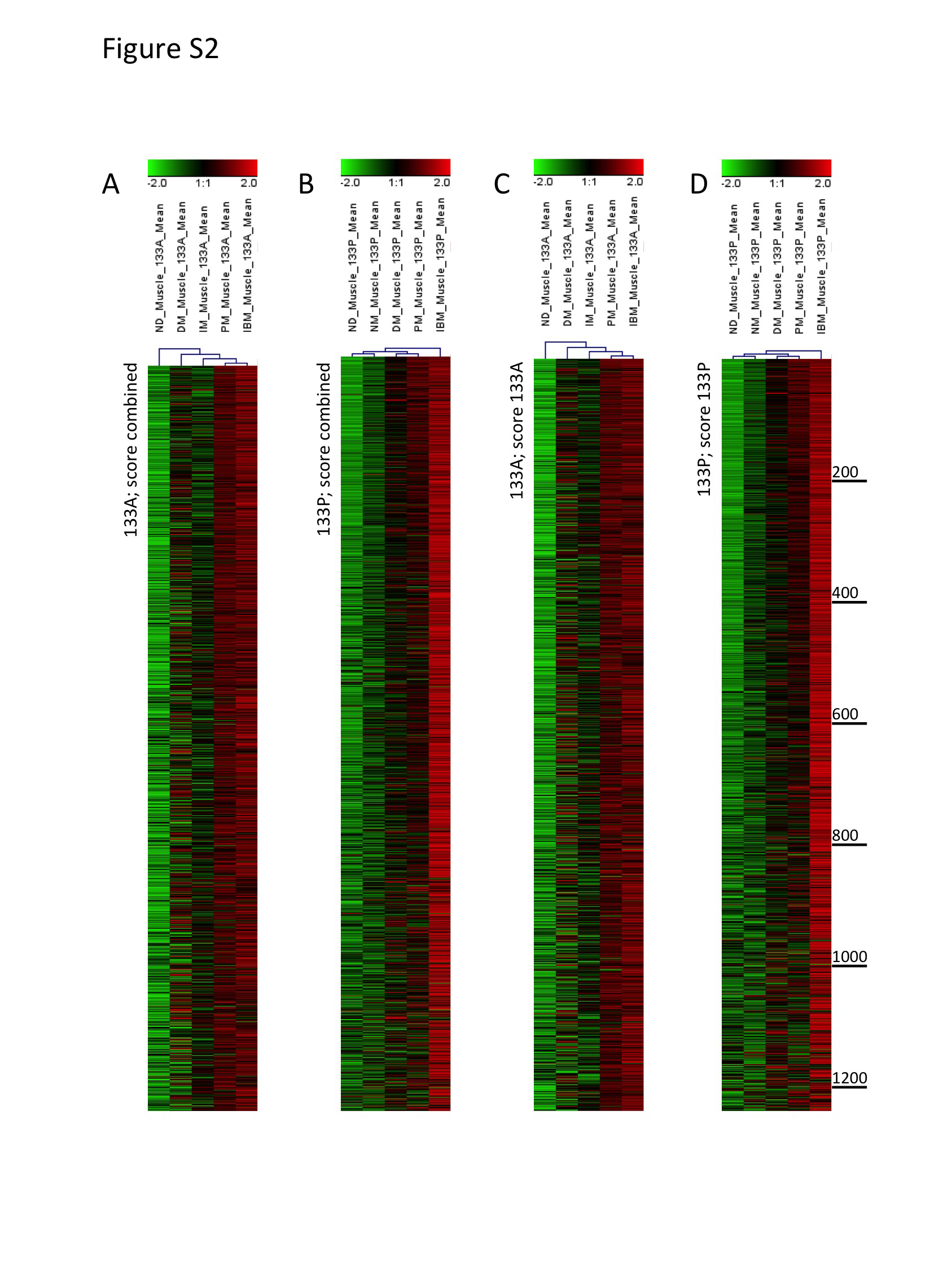

Supplement: Figure S2 — Differences in upregulation of myositis related genes between IBM, PM, DM, NM and IM: All 1209 probesets were sorted by a sum-score for magnitude and frequency of increase in myositis. The heat map presents each disease group by the mean values of the signal intensity in all samples of the group. Combined scoring according to analysis on the 133A and 133P platform demonstrates that the strongest increase is observed in IBM followed by PM and DM, while IM and NM were closest to healthy control. This pattern was observed in 133A samples as well as in 133P samples independently of combined scoring (A and B) or scoring based on each individual platform (C and D). (TIF) [file pone.0104048.s002.tif]

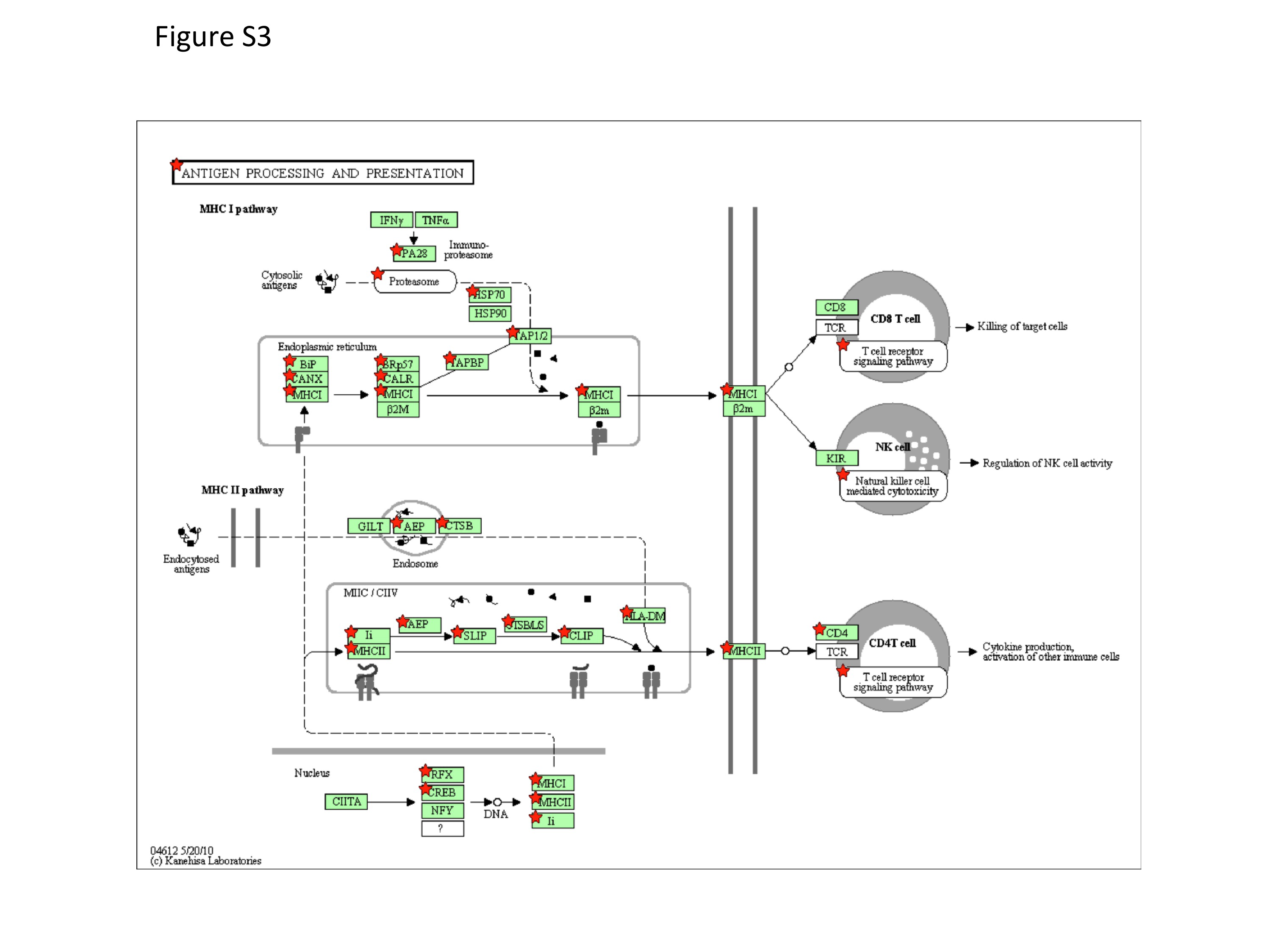

Supplement: Figure S3 — Identification of genes involved in MHC-I and MHC-II antigen processing and presentation pathways: The 1209 probesets upregulated in myositis were uploaded into the DAVID database (http://david.abcc.ncifcrf.gov/) for functional annotation. All genes highlighted with a red star are included in the 1209 probesets. (TIF) [file pone.0104048.s003.tif]

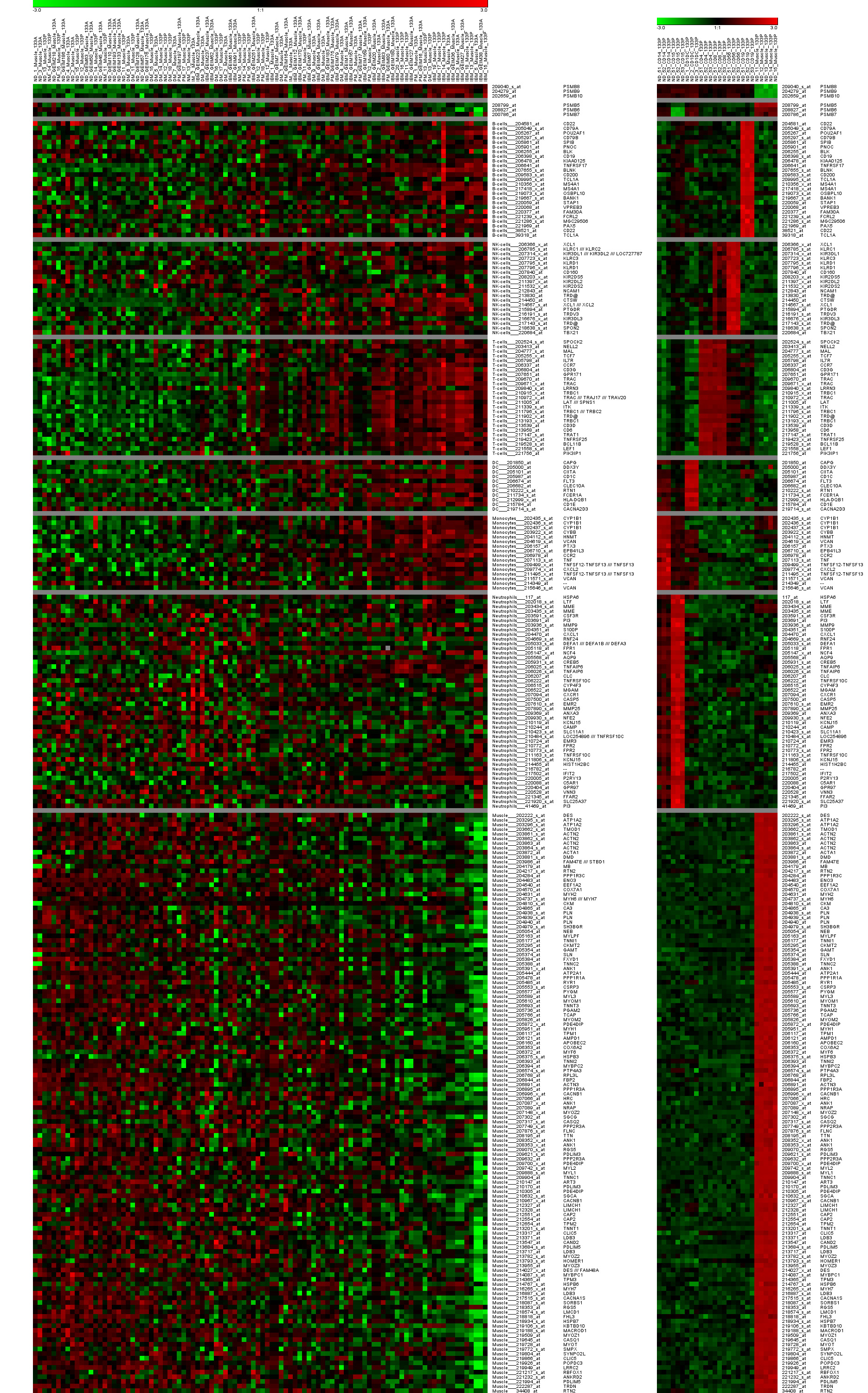

Supplement: Figure S5 — Cell type specific transcripts and corresponding changes of gene expression in myositis: Cell type specific transcripts were determined from transcriptomes of monocytes, neutrophils, CD1+ dendritic cells, T-cells, B-cells, NK-cells and muscle tissue by filtering for cell type specific transcripts with signal level >2000 in the population of interest, <200 in all other populations and a fold change of >20 if possible. In the heatmap on the right side, there is some overlapping expression in the different types of phagocytic cells and in the different lymphocyte populations. CD4+ and CD8+ T-cells do not allow the establishment of a transcript pattern that will distinguish them from other cell types and at the same time will differentiate between these two T-cell subpopulations. In the heatmap on the left side, all myositis transcriptomes were mapped to these marker panels and samples were sorted by intensity of change in the 1209 “myositis genes”. This was performed using the median of log-transformed and z-normalized signals of all 1209 probesets for each sample as a score (myositis score). Sorting myositis samples from the lowest score on the left side (predominantly normal donor samples) to the highest score on the right side (predominantly IBM samples), there is an increase especially of transcripts related to monocytes, dendritic cells and T-cells corresponding to the severity of myositis with a corresponding decrease of muscle specific transcripts. (Figure S5 is also provided as an additional separate jpg-file for further magnification: http://www.charite-bioinformatik.de/supplementary_data/immunoproteasomes/yMDI2dDbwsPIJdDBwNDNwCjLzDgowsDIEK_Figure_S5.jpg). (TIF) [file pone.0104048.s005.tif]
